# The Metabolome in Finnish Carriers of the *MYBPC3*-Q1061X Mutation for Hypertrophic Cardiomyopathy

**DOI:** 10.1371/journal.pone.0134184

**Published:** 2015-08-12

**Authors:** Benedicte Jørgenrud, Mikko Jalanko, Tiina Heliö, Pertti Jääskeläinen, Mika Laine, Mika Hilvo, Markku S. Nieminen, Markku Laakso, Tuulia Hyötyläinen, Matej Orešič, Johanna Kuusisto

**Affiliations:** 1 Hormone laboratory, Aker hospital, Oslo University Hospital, Oslo, Norway; 2 Division of Women and Children’s Health, Department of Pediatric Research, Oslo University Hospital, Oslo, Norway; 3 Helsinki University Central Hospital, Department of Cardiology, Helsinki, Finland; 4 Kuopio University Hospital, Heart Center, Kuopio, Finland; 5 University of Eastern Finland and Kuopio University Hospital, Department of Medicine, Kuopio, Finland; 6 Steno Diabetes Center, 2820 Gentofte, Denmark; 7 VTT Technical Research Centre of Finland, Espoo, Finland; Loyola University Chicago, UNITED STATES

## Abstract

**Aims:**

Mutations in the cardiac myosin-binding protein C gene (*MYBPC3*) are the most common genetic cause of hypertrophic cardiomyopathy (HCM) worldwide. The molecular mechanisms leading to HCM are poorly understood. We investigated the metabolic profiles of mutation carriers with the HCM-causing *MYBPC3-*Q1061X mutation with and without left ventricular hypertrophy (LVH) and non-affected relatives, and the association of the metabolome to the echocardiographic parameters.

**Methods and Results:**

34 hypertrophic subjects carrying the *MYBPC3-*Q1061X mutation, 19 non-hypertrophic mutation carriers and 20 relatives with neither mutation nor hypertrophy were examined using comprehensive echocardiography. Plasma was analyzed for molecular lipids and polar metabolites using two metabolomics platforms. Concentrations of branched chain amino acids, triglycerides and ether phospholipids were increased in mutation carriers with hypertrophy as compared to controls and non-hypertrophic mutation carriers, and correlated with echocardiographic LVH and signs of diastolic and systolic dysfunction in subjects with the *MYBPC3-*Q1061X mutation.

**Conclusions:**

Our study implicates the potential role of branched chain amino acids, triglycerides and ether phospholipids in HCM, as well as suggests an association of these metabolites with remodeling and dysfunction of the left ventricle.

## Introduction

Hypertrophic cardiomyopathy (HCM) is an autosomally inherited myocardial disease occurring in approximately 1 out of 500 individuals [1]. Mutations in the genes encoding mainly cardiac sarcomeric proteins result in ventricular hypertrophy and dysfunctional myocardium. HCM patients have an increased risk of sudden death, and HCM is the most common cause of sudden cardiac death attributable to fatal ventricular arrhythmias in athletes and adolescents. Although many patients remain asymptomatic through life, some develop dyspnea, arrhythmias and end-stage heart failure [1].

Mutations of the myosin-binding protein C (MYBPC) gene often result in truncated proteins, and are the most common genetic cause of HCM worldwide [2]. In Finland, a novel founder mutation of the cardiac myosin-binding protein C gene (*MYBPC3*-Q1061X) has been identified in Finnish HCM patients, which leads to a truncated protein lacking the binding sites for myosin and titin [3,4] (S1 Fig). This mutation accounts for approximately 11% of HCM in Finnish patients, and is the most prevalent HCM-causing mutation in Finland with a penetrance of approximately 78% [5]. Because *MYBPC3* mutations in previous studies have been reported to have a rather favorable prognosis, it may be even more common [6,7]. Generally, the phenotype of subjects with *MYBPC3* mutations is variable, and cardiac hypertrophy usually does not manifest until early adolescence with penetrance increasing with age and peaking at approximately 60 years of age [8–10]. The molecular mechanisms leading to HCM in mutation carriers, such as asymmetric hypertrophy, myofibrillar disarray and fibrosis, are poorly understood.

Metabolomics is the global study of metabolites, such as lipids, sugars and amino acids, in cells, tissues and biofluids. The metabolome is sensitive to various pathogenically relevant factors including genetic variation, diet, age, immune system status and gut microbiota [11–15]. Metabolomics is therefore a powerful tool for the characterization of complex phenotypes affected by genetic and environmental factors as well as the interactions thereof [16]. Metabolomics studies in HCM and other cardiovascular diseases are of particular interest, both to better understand the pathophysiology and to identify novel biomarkers [17]. Since cardiovascular diseases are often characterized by long prodromal periods, with clinical symptoms occurring rather late in the pathogenesis, it is important to identify individuals at high risk in the prodromal phase when intervention would be most optimal. Several different metabolite profiling methods have been developed, which allow for extensive and quantitative investigation of a large amount of different metabolites [18]. To our knowledge, no metabolomics studies on HCM in humans have so far been reported.

We aimed to investigate the metabolic profiles of carriers of the *MYBPC3*-Q1061X mutation with and without left ventricular hypertrophy (LVH), and compare these profiles to those of subjects with neither the mutation nor LVH. All subjects were assessed with comprehensive echocardiography including tissue Doppler imaging. Plasma samples were analyzed with two analytical platforms for metabolomics, with broad analytical coverage of molecular lipids and polar metabolites.

## Methods

### Subjects

A total of 73 genotyped subjects from families carrying the *MYBPC3*-Q1061X mutation were recruited from the Helsinki and Kuopio University Hospital regions. Mutation carriers diagnosed with clinical HCM (maximum wall thickness of left ventricle (MWT) ≥ 13 mm on echocardiography) numbering 34 patients were included in the G+/LVH+ group. The G+/LVH- group included 19 mutation carriers without hypertrophy (MWT < 13 mm), and the control group included 20 subjects from the same families with neither the mutation nor LVH. Exclusion criteria were age (<18 years) and pregnancy. The cardiac phenotype of the subjects was characterized by anthropometrics, blood samples and echocardiography. The blood samples were collected in the fasting state (12 hours) and immediately centrifuged at 3200 G for 10 minutes at 4°C. Plasma was separated and stored at -70°C (Kuopio) and -80°C (Helsinki). The local ethics committees of the University Hospitals of Helsinki and Kuopio in Finland approved the study protocol (96/2008). The study is in agreement with the principles outlined in the Declaration of Helsinki. All patients gave written consent.

### Echocardiography

To assess cardiac structural changes and cardiac function, GE Vivid 7 ultrasound equipment with an M4S probe at the University Hospitals of Helsinki and Kuopio were used. Echocardiography was performed by experienced clinical cardiologists (MJ, PJ, JK). Measurements were analyzed offline with EchoPac software (GE Vingmed, version 10.0.1, Norway) by the same observer (MJ) blinded to clinical and genetic data at the Helsinki University Central Hospital.

Conventional measurements of cardiac anatomy and function were performed with M-mode, 2D and pulsed-wave Doppler according to guidelines [19]. Biplane Simpson method in the apical four- and two-chamber views was used to measure left ventricular ejection fraction (LVEF). Parasternal short axis views were used to measure maximum wall thickness (MWT). If apical hypertrophy was suggested by imaging an apical view was employed. Presence of systolic anterior motion (SAM) and left ventricular outflow tract (LVOT) obstruction, defined as a gradient of ≥ 30 mmHg measured at rest, were recorded. Left ventricular mass (LV mass) was calculated by the formula: 0.80 (1.04 x [PWT + SWT+LVEDD]^3^ –LVEDD^3^) + 0.6), where PWT and SWT is posterior and septal wall thickness, respectively, and LVEDD is left ventricular internal diameter at diastole [20].

Tissue Doppler imaging (TDI) velocities of systolic (Sm) and early diastolic motion (Em) were measured from the septal and lateral mitral valve annulus.

### Metabolomic analysis

Two analytical platforms for metabolic profiling were applied to all samples: (a) platform for global profiling of small, polar metabolites, based on comprehensive two-dimensional gas chromatography coupled with time-of-flight mass spectrometry (GC×GC-TOFMS), covering molecules such as sugars, sterols, amino acids and various organic acids, including free fatty acids and ketoacids; (b) global lipidomics platform based on ultra performance liquid chromatography coupled to quadrupole time-of-flight mass spectrometry (UPLC-QTOFMS), covering various molecular lipids such as phospholipids, triglycerides, sphingolipids and neutral lipids. Both platforms have been extensively described [21,22], and are also described in the S1 Methods. The raw data from UPLC-QTOFMS platform were processed with MZmine 2 [23], while the GC×GC-TOFMS data were processed with the Guineu software [21].

A total of 699 molecular lipids and 1603 small polar metabolites were detected, of which 238 lipids and 215 metabolites were identified. Only lipids and metabolites identified and found in at least 70% of the samples were used in the statistical analysis.

### Cluster analysis

The lipidomics and metabolomics data were scaled with zero mean and unit variance to make the profiles comparable with each other, and Bayesian model-based clustering was applied on the scaled data to group metabolites with similar profiles across all samples. The clustering was performed using the mclust [24] method, implemented in R [25] in the package “metadar” [26]. In mclust, the observed data are viewed as a mixture of several clusters and each cluster comes from a unique probability density function. A number of clusters in the mixture, together with the cluster-specific parameters that constrain the probability distributions, will define a model, which can then be compared with others. The clustering process selects the optimal model and determines the data partition accordingly.

### Statistical analyses and linear mixed models

Statistical analyses were performed with R version 2.15.0 for Windows (R Development Core Team (2011), R Foundation for Statistical Computing, Vienna, Austria) and IBM SPSS Version 19.0 (IBM Corp. Released 2010. IBM SPSS Statistics for Windows, Version 19.0. Armonk, NY: IBM Corp.). Most analyses and visualization in R were performed using the “metadar” package for metabolomics data analysis [26]. Clinical, echocardiographic and individual metabolite level data between the study groups were compared with the independent samples T-test or Fisher’s exact test for 2 groups and for 3-groups we employed one-way analysis of variance (ANOVA) with pairwise comparison adjusted with the Bonferroni correction or Tukey’s range test. The average within-cluster metabolite profiles between the three groups were analyzed with one-way ANOVA. Analysis of covariance (ANVOCA) was performed by adjusting the data for age and gender, and then performing one-way ANOVA on the adjusted data. Individual metabolite levels were visualized using beanplots [27], implemented in the ‘beanplot’ R package. Beanplots provide information on the mean metabolite level within each group, the density of the data-point distribution, as well as show individual data points. To assess the association between metabolic profile and echocardiographic parameters in mutation carriers with and without hypertrophy, correlation analyses using Spearman product-moment correlation for pairwise complete data were performed in R, as implemented in “metadar” [26]. Scatterplots and linear regression between metabolite levels and echocardiographic parameters were performed in SPSS. All tests were assessed with a significance level of *p*≤ 0.05.

## Results

### Clinical characteristics and echocardiography

The baseline clinical characteristics are presented in Table 1. There were more male subjects in the pooled G+ mutation carrier group compared to the control group. The subjects in the G+ group were only asymptomatic or mildly symptomatic (New York Heart Association Functional Classification class I-II). Some of the subjects in both study groups had hypertension, and a few of the subjects in the G+ group had atrial fibrillation (AF). There was no difference in the body mass index, heart rate, blood pressure or lipid levels between the control and G+ groups. There was no significant difference in age between the control and G+ subjects, but in the subgroups analysis, G+/LVH- subjects were younger than subjects in other two groups (data not shown). Baseline data is listed in S1 Dataset.

**Table 1:** Clinical characteristics of the *MYBPC3*-Q1061X mutation carriers (G+) and control group.

Baseline	Control (N = 20)	G+ (N = 53)	p-value
Male	5/25%	29/55%	0.035
Age (years)	46 ± 17	46 ± 15	0.906
Body mass index	25 ± 4	26 ± 5	0.356
Hypertension	5/25%	15/28%	1.000
Diabetes	2/10%	4/8%	0.663
Atrial fibrillation (N/%)	0/0%	4/8%	0.57
NYHA			
I		49/92%	
II		4/8%	
Heart rate (bpm)	63 ± 8	62 ± 10	0.878
Systolic blood pressure (mmHg)	133 ± 13	127 ± 16	0.167
Diastolic blood pressure (mmHg)	78 ± 9	77 ± 9	0.690
Cholesterol (mmol/l)	4.4 ± 0.9	4.5 ± 0.8	0.606
LDL (mmol/l)	2.6 ± 0.7	2.6 ± 0.8	0.970
HDL (mmol/l)	1.4 ± 0.3	1.4 ± 0.5	0.789
Triglycerides (mmol/l)	0.9 ± 0.6	1.2 ± 0.7	0.095

Values are presented as mean ± SD or count and percentage, with p-values for T-test or Fisher’s exact test between groups. NYHA = New York Heart Association functional class, LDL = low-density lipoprotein, HDL = high-density lipoprotein.

Echocardiographic data in control, G+/LVH- and G+/LVH+ groups are shown in Table 2. Left ventricular dimensions and ejection fraction (LVEF) were within normal range in all three groups. By definition, MWT and LV mass were higher in hypertrophic subjects compared to non-hypertrophic subjects. Left atrial size was larger in the G+/LVH+ group compared to other two groups. There was no difference in LVOT gradient between the three groups, but 7 patients of the G+/LVH+ groups had systolic anterior motion of the mitral valve apparatus (SAM) and two had LVOT obstruction (LVOT gradient > 30 mmHg at rest). Mitral valve E velocity did not differ between the study groups. TDI septal and lateral annular Sm was diminished in the G+/LVH+ group indicating systolic dysfunction despite preserved LVEF. In the G+/LVH+ group, early diastolic velocities at the lateral and septal mitral annulus were significantly lower compared to other two groups, consistent with diastolic dysfunction typical of HCM. The G+/LVH- group had higher TDI lateral Em compared to the control group, presumably because the G+/LVH- group was younger than the control group. Otherwise there were no significant differences in baseline echocardiographic variables between the G+/LVH- and control groups (Table 2). Echocardiographic data is listed in S2 Dataset.

**Table 2:** Echocardiographic parameters for the study subjects.

Echocardiography	Control (N = 20)	G+/LVH- (N = 19)	G+/LVH+ (N = 34)	p-value
LVEDD (mm)	49 ± 5	46 ± 6	46 ± 8	0.352
LVESD (mm)	28 ± 4	30 ± 6	30 ± 9	0.838
LVEF (%)	61 ± 5	61 ± 5	61 ± 11	0.997
MWT (mm)	10.3 ± 1.0	10.6 ± 1.4	20.4 ± 5.9 ^a^ ^,^ ^b^	< 0.001
LV Mass (g)	154 ± 35	148 ± 55	258 ± 100 ^a^ ^,^ ^b^	< 0.001
LAD (mm)	33 ± 5	34 ± 5	39 ± 8 ^a^ ^,^ ^b^	< 0.001
LVOT gradient (mmHg)	7.1 ± 2.9	5.7 ± 1.8	8.9 ± 12.0	0.398
MV E velocity (m/s)	0.8 ± 0.1	0.8 ± 0.2	0.7 ± 0.2	0.100
TDI Lateral Sm (cm/s)	9.0 ± 3.1	9.9 ± 2.5	6.5 ± 1.9 ^a^ ^,^ ^b^	< 0.001
TDI Septal Sm (cm/s)	8.1 ± 2.0	7.9 ± 1.4	6.6 ± 1.7 ^a^ ^,^ ^b^	0.002
TDI Lateral Em (cm/s)	12.2 ± 4.6	15.8 ± 4.1^c^	9.1 ± 3.9 ^a^ ^,^ ^b^	< 0.001
TDI Septal Em (cm/s)	11.3 ± 3.8	11.9 ± 2.6	6.4 ± 2.8 ^a^ ^,^ ^b^	< 0.001

The parameters are referred to as mean ± SD. Abbreviations: LVEDD left ventricular (LV) internal diameter in diastole, LVESD LV internal diameter in systole, LVEF left ventricular ejection fraction, MWT maximum wall thickness, LV Mass left ventricular mass, LAD left atrium diameter, LVOT gradient left ventricular outflow tract gradient, MV E velocity mitral valve E-wave peak velocity, TDI Lateral Sm TDI peak systolic velocity at lateral mitral annulus, TDI Septal Sm TDI peak systolic velocity at septal mitral annulus, TDI Septal Em TDI peak early diastolic velocity at septal mitral annulus, TDI Lateral Em peak early diastolic velocity at lateral mitral annulus. P-values are given as one-way ANOVA. Pairwise comparisons with Bonferroni corrected p-values are labeled:

^a^P<0.05 between G+/LVH+ and control group,

^b^P<0.05 between G+/LVH+ and G+/LVH-,

^c^P<0.05 between G+/LVH- and control group.

### Association of the global metabolome with the MYBPC3-Q1061X mutation and LVH

**Fig 1:**
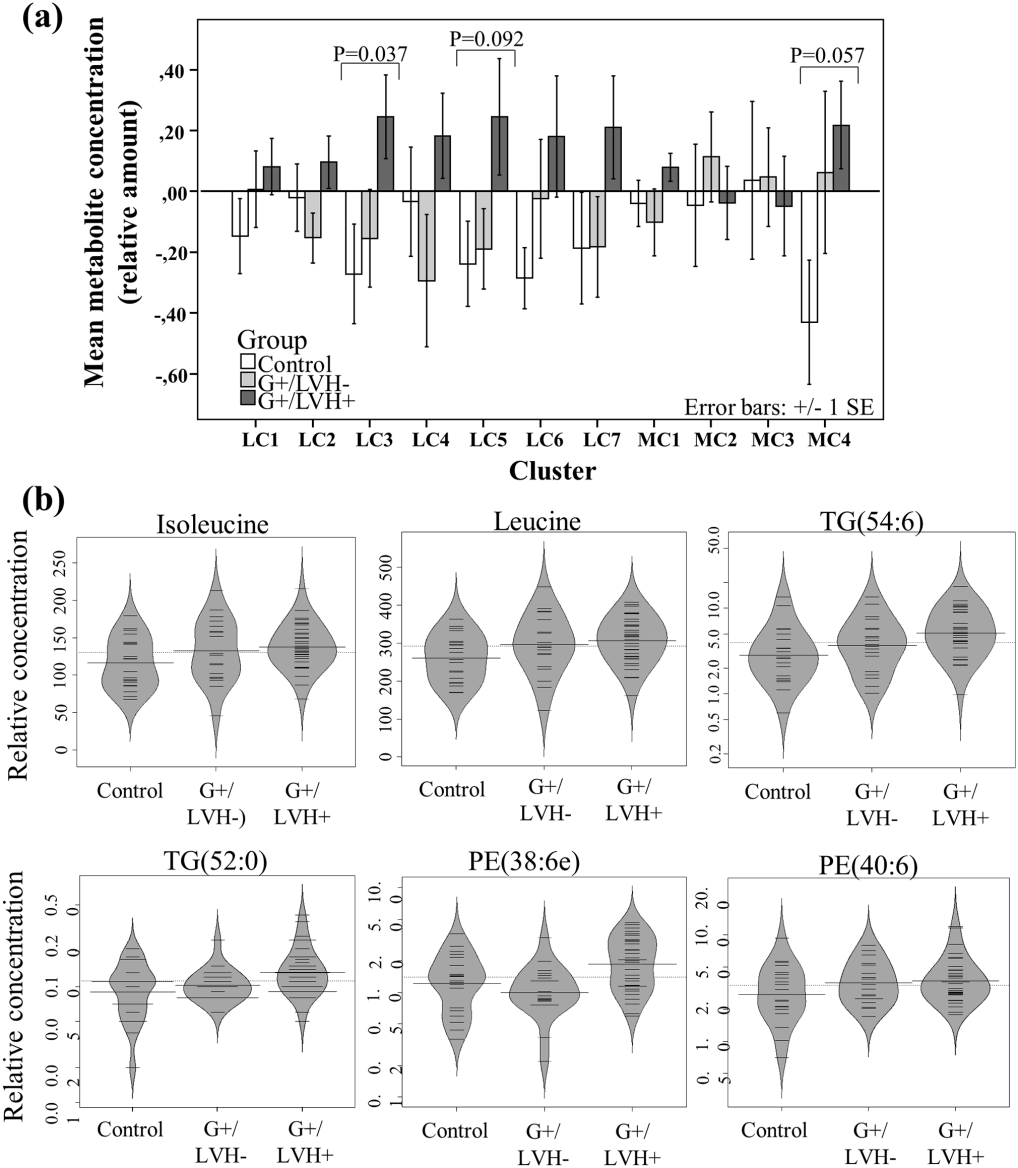
Metabolomic profiles across the three study groups. (a) Mean metabolite levels within each cluster for the three groups. The error bars show standard error of the mean (SEM), and cluster LC3 is the only significant cluster. Nominal p-values are shown (one-way ANOVA). (b) Profiles of selected representative metabolites from different clusters in all three groups. The metabolite levels are shown as beanplots [27], which provide information on the mean level (solid line), individual data point (short black lines), and the density of the distribution.

Following the data processing and filtering as described in Methods, a total of 86 polar metabolites and 238 molecular lipids were included in the data analysis. Metabolite-data are listed in S3 Dataset, as is lipid-data (S4 Dataset). Since many metabolites are co-regulated, it cannot be assumed that the profiles of all measured metabolites are independent [28]. Therefore, all lipids and metabolites were first surveyed by clustering of the data into subsets using Bayesian model-based clustering [24]. The identified lipids and polar metabolites were decomposed into seven and four clusters, respectively (LC1-LC7, MC1-MC4; Table 3). The division of lipid clusters reflects different lipid functional and structural groups, while the metabolite profiles are decomposed into three small clusters and one large cluster comprising different metabolite functional groups. When comparing the three groups (one-way ANOVA), one of the lipid clusters reached significance level (LC3) while one lipid (LC5) and one metabolite (MC4) cluster were marginally significant (Fig 1a).

**Table 3:** Description of metabolite clusters from the lipidomics (LC) and metabolomics (MC) platforms.

Cluster	Cluster-size	Description	Examples of metabolites
LC1	46	Mainly PCs, but also PEs and TGs	PC(36:4), PE(34:1), TG(53:2)
LC2	86	Mainly PCs with ether-linkages and PEs, but also ChoEs, SMs, TGs	PC(38:5e), PE(40:3), ChoE(18:2), PC(40:4), SM(d18:1/22:1)
LC3	36	PUFA-containing PC and PE (plasmalogens) and PUFA-containing TGs	PC(38:6), PE(40:6), TG(56:7)
LC4	10	LysoPCs	LysoPC(18:2)
LC5	21	Mainly saturated TGs and 1–3 double bonds, longer chains than LC6	TG(50:1), TG(50:3), TG(54:0)
LC6	19	Saturated TGs and TGs with 1–3 double bonds, shorter chains than LC3, LC5 and LC7	TG(42:1), TG(47:0), TG(48:3)
LC7	20	PUFA-containing TGs, long chains	TG(51:5), TG(54:3), TG(54:7)
MC1	69	Diverse	4-methyl-2-oxovaleric acid, valine, hexanoic acid
MC2	11	Carboxylic acids	Linoleic acid, oleic acid
MC3	4	Carboxylic acids	Hexadecanoic acid, phosphoric acid
MC4	2	Branched-chain amino acids	Leucine and isoleucine

Abbreviations: PC phosphatidylcholine, PE phosphatidylethanolamine, TG triglyceride, ChoE cholesteryl ester, SM sphingomyelin, PUFA polyunsaturated fatty acid, lysoPC lysophosphatidylcholine.

The lipid cluster LC3 which mainly includes long-chain polyunsaturated fatty acid (PUFA) containing triglycerides (TGs), and phospholipids (plasmalogens) was significantly elevated in HCM patients (*p* = 0.037), especially when compared to the control group (*p* = 0.047, Tukey’s range test). This indicates that increased levels of TGs and phospholipids with a high degree of double bonds are associated with HCM caused by *MYBPC3*-Q1061X. The remaining lipid clusters also tended to be higher in HCM patients, although none of these differences were statistically significant.

One of the smaller clusters comprising polar metabolites, MC4, contains two branched chain amino acids (BCAAs) leucine and isoleucine, while two other small clusters contain carboxylic acids. The large cluster contains various metabolites, among others 4-methyl-2-oxovaleric acid, a catabolic product of leucine, and several organic acids involved in the citric acid cycle, including α-ketoglutaric acid and malic acid.

### Association of molecular lipids and polar metabolites with the MYBPC3-Q1061X mutation and LVH

When analyzing the lipids at the molecular level (one-way ANOVA), several molecular lipids were elevated in the G+/LVH+ group **(** Table 4). Out of the 238 lipids, 28 were different between the groups. Several TGs were increased in the G+/LVH+ group compared to the control group, and some also to the G+/LVH- group. These TGs all had long-chain, even-numbered (54–58 carbons) fatty acids and were mostly polyunsaturated (4–7 double bonds). The only TG which was decreased in the G+/LVH+ group as compared to both the control group and G+/LVH- group was a triglyceride with an odd number of acyl carbons; TG(53:7) (*p* = 0.024 and *p* = 0.026, respectively). Most of the TGs were marginally increased in the G+/LVH- group compared to the control group, although none of the differences was statistically significant. The two ether phospholipids PE(38:6e) and PE(38:7e) were increased in the G+/LVH+ group compared to the control group.

**Table 4:** Mean metabolite-levels for significant molecular lipids and polar metabolites.

Metabolite-name	Control (N = 20)	G+/LVH- (N = 19)	G+/LVH+ (N = 34)	P-value
PE(38:6e)	1.46±0.86	1.17±0.68	2.18±1.19^a^ ^,^ ^b^	0.001
PE(38:6e)	2.93±1.07	2.39±0.64	3.56±1.32^b^	0.002
PE(40:8e)	0.63±0.23	0.55±0.18	0.79±0.28^b^	0.003
PE(38:7e)	1.17±0.57	1.04±0.67	1.69±0.83^a^ ^,^ ^b^	0.004
PE(36:4e)	1.14±0.34	0.93±0.24	1.33±0.50^b^	0.004
PE(40:8e)	1.02±0.33	0.95±0.29	1.22±0.32^b^	0.008
PE(38:5e)	6.65±2.04	5.45±1.93	7.62±2.86^b^	0.011
PE(p16:0/22:6)	2.39±0.72	2.31±0.69	2.92±0.95^b^	0.017
PE(40:6)	3.97±1.40	3.93±1.51	5.40±2.70^b^	0.019
PE(40:7e)	2.60±0.72	2.31±0.92	3.03±0.99^b^	0.020
PE(40:4)	10.39±3.15	10.17±2.53	12.09±3.12	0.041
PC(38:7e)	0.55±0.18	0.46±0.13	0.61±0.21^b^	0.023
PC(38:6e)	0.86±0.21	0.83±0.21	1.00±0.26^b^	0.026
PC(p16:0/20:4)	5.88±1.93	5.86±1.66	7.11±2.12	0.032
PC(30:3)	0.31±0.10	0.26±0.07	0.34±0.13^b^	0.037
PC(34:2e)	3.34±1.32	2.86±0.85	3.98±1.89^b^	0.038
TG(58:5)	0.07±0.03	0.08±0.04	0.11±0.04^a^ ^,^ ^b^	0.004
TG(56:4)	0.16±0.08	0.17±0.09	0.28±0.19^a^ ^,^ ^b^	0.005
TG(53:7)	0.12±0.03	0.12±0.04	0.09±0.03^a^ ^,^ ^b^	0.008
TG(56:5)	2.20±1.01	2.19±0.83	2.90±1.12^a^ ^,^ ^b^	0.017
TG(52:0)	0.10±0.05	0.11±0.04	0.15±0.08^a^	0.023
TG(54:5)	6.47±3.56	7.68±3.70	9.49±4.52^a^	0.031
TG(54:6)	3.74±3.24	4.65±3.34	6.15±3.77^a^	0.048
TG(56:6)	7.62±5.18	7.25±3.23	9.84±3.81	0.049
ChoE(20:5)	7.39±4.44	7.40±7.44	10.85±5.58	0.048
LysoPC(20:5)	0.63±0.35	0.63±0.43	0.91±0.57	0.050
SM(d18:1/16:0)	82.32±12.13	76.42±10.67	85.43±13.75^b^	0.050
Glutamic acid	238.16±86.99	191.38±82.56	269.83±94.89^b^	0.012
Threonine	259.55±91.03	264.79±113.89	200.79±82.29	0.025
2-Butenedioic acid	39.08±19.03	32.44±14.97	46.68±20.28^b^	0.027
4-Methyl-2-oxovaleric acid	397.08±143.47	409.86±104.14	492.86±106.10^a^ ^,^ ^b^	0.007
Valine	338.98±45.91	360.77±60.62	380.53±50.02^a^ ^)^	0.021
Leucine	260.98±61.53	296.30±80.16	307.23±60.12^a^	0.050
Isoleucine	115.79±33.76	132.20±41.73	137.47±29.94	0.085*

The metabolite-levels are referred to as mean±SD. Abbreviations: PE phosphatidylethanolamine, PC phosphatidylcholine, TG triglyceride. P-values are given as one-way ANOVA. Pairwise comparisons with Tukey’s range test corrected p-values are labeled:

^a^P<0.05 between G+/LVH+ and control group, given as Tukey’s range test.

^b^P<0.05 between G+/LVH+ and G+/LVH-, given as Tukey’s range test.

*Not significant

When analyzing individual polar metabolites, six were different between the groups (Table 4). All three BCAAs were increased in the G+/LVH+ group. Valine was 1.12-fold higher compared to the control group and 1.05-fold compared to the mutation carriers in the G+/LVH- group (*p* = 0.021). Leucine and its metabolic product, 4-methyl-2-oxovaleric acid (ketoleucine), had even higher concentrations in the G+/LVH+ group compared to the other groups (*p* = 0.05 and *p* = 0.007, respectively). Leucine was increased 1.18-fold compared to the control group (*p* = 0.041), and 4-methyl-2-oxovaleric acid was increased 1.24-fold (*p* = 0.013) and 1.2-fold (*p* = 0.041) compared to the control group and mutation carriers without LVH, respectively. Because of the difference in age and gender between the groups, we also performed an ANCOVA adjusted for these two variables. Valine and leucine were then only marginally different (*p* = 0.060 and *p* = 0.059), while 4-methyl-2-oxovaleric acid was not significant (S1 Table). Isoleucine was also increased in the G+/LVH+ group, by 1.19-fold compared to the control group and 1.04-fold compared to mutation carriers without LVH, although the differences were not statistically significant.

Selected lipids and polar metabolites are shown in Fig 1b.

### Correlation analysis of metabolomic variables to echocardiographic measurements

**Fig 2:**
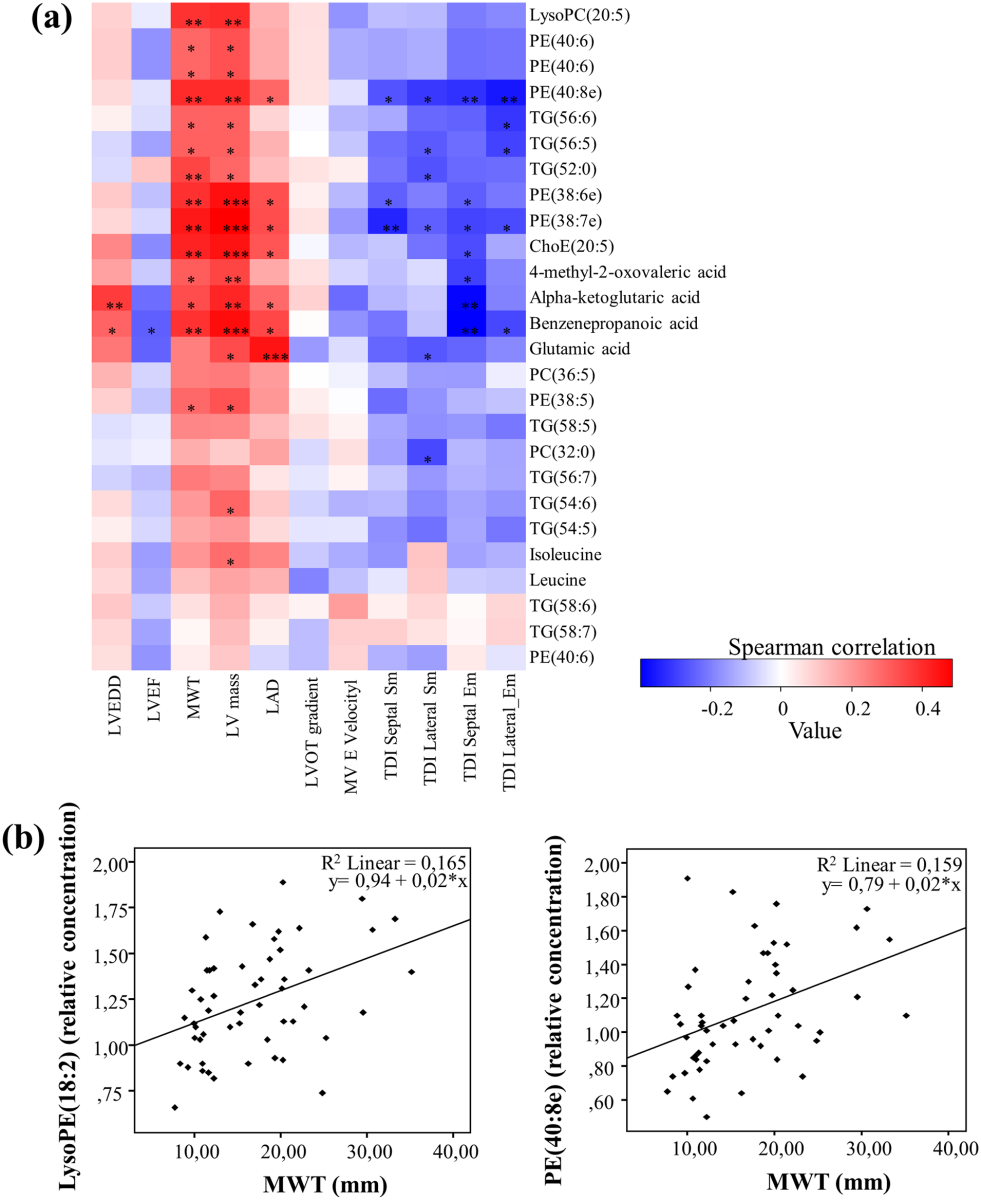
Correlation heatmap and scatterplots. (a) Heatmap of Spearman product-moment correlation coefficients between metabolites and echocardiographic parameters for pooled G+/LVH- and G+/LVH+ groups. The colors represent the correlation, with red being more positive and blue more negative. Significance is given as *(*p*<0.05), **(*p*<0.01) and ***(*p*<0.001). (b) Scatterplots showing the relationship between lysoPE(18:2) and MWT (left), and PE(40:8e) and MWT (right).

Spearman correlation coefficients for the relationship between the metabolites and the echocardiographic parameters were obtained for the pooled G+/LVH- and G+/LVH+ groups (Fig 2). In subjects with the *MYBPC3*-Q1061X mutation, 4-methyl-2-oxovaleric acid, alpha-ketoglutaric acid, benzenepropanoic acid and glutamic acid were positively correlated with several of the echocardiographic variables, such as LVEDD, MWT, LV mass and LAD. The lysophospholipids, phospholipids and triglycerides were positively correlated with MWT and LV mass. Several of the lipids were negatively correlated to TDI septal Sm, TDI lateral Sm, TDI septal Em and TDI lateral Em, with the PE plasmalogens being significantly correlated. The odd-numbered triglyceride TG(53:7) showed an opposite correlation pattern as compared to the other TGs.

## Discussion

### Principal findings

We have shown that the patients with HCM attributable to the Q1061X mutation of *MYBPC3* display elevated levels of several triglycerides and amino acids including BCAAs, as well as some phospholipids, compared to control subjects without LVH. Although we did not detect any significant metabolite-differences between mutation carriers without hypertrophy and the control group, there is a trend of several of the metabolites in the non-hypertrophic mutation carrier group in the same direction as the metabolites in the hypertrophic group. In the pooled group analysis including all subjects with the *MYBPC3-*Q1061X mutation, several metabolites, including several TGs, ether PEs and lysophospholipids were significantly correlated with indices of LV remodeling such as hypertrophy, LV mass, and systolic and diastolic dysfunction, indicating a shift in the metabolic profile with manifest hypertrophic cardiomyopathy.

### In the context of current literature

To our knowledge, no metabolomics studies of HCM in humans have so far been reported. In earlier metabolomics studies using a similar design on dilated cardiomyopathy (DCM), potential biomarkers such as steroid metabolites, glutamine, threonine and histidine were found to be diminished and TCA cycle intermediates and lipid β-oxidation products were found to be increased in DCM patients compared to control individuals [29]. We have previously reported that odd-chain triglycerides are diminished in patients with DCM attributable to the mutations in the lamin A/C gene (*LMNA*) [30]. In hamster models with a pathophysiology similar to human DCM, several metabolites involved in glycolysis, pentose phosphate pathway and TCA cycle, together with triglycerides, were decreased as compared to the control animals [18]. These studies suggest altered energy production and tissue remodeling in DCM. Comparing the metabolomic changes in DCM to the results of this study on HCM, both exhibit a decrease in glutamate and increase in threonine levels, but in contrast the levels of triglycerides are decreased in DCM and increased in HCM.

### Possible mechanisms

In our study, levels of several TGs with even-numbered chains were increased in hypertrophic mutation carriers. The levels of these TGs correlated with several echocardiographic variables such as increased wall thickness and reduced systolic and diastolic function. Substrates for myocardial energy production in humans are variable throughout life. While the healthy adult heart primarily oxidize long-chain fatty acids as energy supply [31], the hypertrophic and failing heart shifts towards glucose and lactate metabolism, which is also seen in conditions such as ischemia, hypothyroidism, hypoxia, diabetes and athrophy [32]. This transition from oxidative fatty acid metabolism to glucose metabolism in cardiac hypertrophy is thought to be associated with the pathological remodeling of the heart [33]. A Finnish study of patients with HCM attributable to the Asp175Asn mutation in the α-tropomyosin gene revealed increased myocardial oxidative metabolism and free fatty acid metabolism in HCM patients with mild LVH, which decreased with advanced LV hypertrophy [34]. The increased levels of circulating TGs in HCM patients in this study might represent a shift in myocardial metabolism substrates.

Phospholipids are major components in heart tissue, and patients with HCM exhibited elevated levels of several ether phospholipids compared to mutation carriers without LVH. Earlier animal studies on hamsters with pathophysiology similar to human DCM [18] and rats with aortic constriction-induced LVH from birth [35] have shown contradictory results on phospholipid composition. Although these remodeling processes probably are different from that of HCM, they indicate a dysregulation of membrane phospholipid homeostasis which may contribute to the development of several cardiomyopathies [36].

In addition to increased lipid levels, HCM patients displayed increased levels of the BCAAs valine and leucine, which are catabolized in non-hepatic tissue, such as cardiac muscle [37]. We found that leucine clusters with isoleucine, meaning they show a similar concentration-pattern among the subjects, while 4-methyl-2-oxovaleric acid clusters together with valine in the larger MC1 cluster. BCAAs are converted into branched-chain α-keto-acids (BCKA) and further into propionyl-CoA by the BCKA dehydrogenase complex (BCKD), before it is degraded into acetyl-CoA or succinyl-CoA and enters the citric acid cycle. The BCKD complex must be dephosphorylated by a protein phosphatase, PP2C, in order to be active [38]. Studies show a high expression of mitochondrial PP2C (PP2Cm) in cardiac muscles in zebra-fish embryos and adult mice, with stress being an important regulator, as demonstrated by significantly reduced expression of both PP2C mRNA and protein in hypertrophic and failing hearts [39]. This reduction leads to elevated plasma levels of BCAA and BCKA in PP2Cm-deficient mice [38].

Morpholinos employed to suppress expression of PP2Cm in developing zebra-fish embryos resulted in dose-dependent development defects, contractile dysfunction and heart failure [39]. BCAAs are also efficient activators of the mTOR (mammalian target of rapamycin) pathway, which regulates protein synthesis. A local elevation of BCAA concentrations may lead to chronic induction of mTOR, promoting cardiac hypertrophy by altering insulin sensitivity [37]. The early development of HCM is characterized by reduced energy efficiency as shown previously by diminished PCr/ATP ratio and myocardial external efficiency even in carriers of HCM mutations without hypertrophy [40,41]. The *MYBPC3*-Q1061X mutation most likely results in haploinsufficiency leading to contractile impairment and impaired energy utilization [42,43]. The LV septum requires most energy in contraction and Ashrafian et al. have hypothesized that this chronic energy depletion leads through myocyte dysfunction, AMP-activated protein kinase activation and increase in cytosolic Ca^2+^ into the characteristic asymmetric septal hypertrophy most commonly seen in HCM [44].

This is the first study to our knowledge to incorporate comprehensive metabolomics analysis and modern echocardiographic data in carriers of a single HCM-causing mutation. The metabolomic profile of increased TGs and BCAAs in HCM patients might indicate a change in energy metabolism similar to that observed in heart failure and hypertrophy due to other causes. The observed trend of these metabolites in the G+/LVH- group, in the same direction as in the HCM patients, might indicate that some changes in metabolism occur already before clinical hypertrophy, despite the lack of significance. Metabolomics might aid in finding non-hypertrophic mutation carriers who are at risk of developing HCM.

### Limitations of the study

Even though the study population was relatively small and heterogeneous, and some HCM patients had hypertension, all participants with HCM had the *MYBPC3-*Q1061X mutation primarily responsible for hypertrophic changes. Also, because the metabolome is sensitive to various changes unrelated to the disease process in question, we tried to minimize these effects with cluster analysis, use of control subjects from the same families to minimize genetic differences, and age-adjustment of comparisons. The study subjects had normal Finnish diet. Finally, although the *MYBPC3*-Q1061X mutation is rare outside Finland, current literature suggest that most of the known *MYBPC3* mutations lead to similar haploinsufficiency through truncated proteins, which are not detectable in the myocardium [42,43]. Therefore, the presented metabolic changes may not just be specific to the *MYBPC3*-Q1061X mutation, but may represent changes common to other *MYBPC3* mutations resulting in truncated proteins.

## Conclusions

In conclusion, we have identified several metabolic changes that appear to be associated with HCM attributable to the *MYBPC3*-Q1061X mutation. Especially the branched chain amino acids valine and leucine, 4-methyl-oxovaleric acid and PUFA-containing long chain TGs tend to show an increasing trend between the three groups, with significant differences between the G+/LVH+ and the control groups. Possible clinical implications for the findings in this study may include the use of the elevated levels of metabolites as biomarkers of the LV remodeling process in HCM, but more studies are needed to investigate the temporal relationship between the levels of metabolites and echocardiographic findings in LV remodeling.

## Supporting Information

S1 Dataset Baseline data. Subject-index is listed in the first row, group in the second row, and baseline-data listed in separate rows.
(XLSX)

S2 Dataset Echocardiographic parameters. Subject-index is listed in the first row, group in the second row, and echocardiographic parameters listed in separate rows.
(XLSX)

S3 Dataset Metabolite data. Subject-index is listed in the first row, group in the second row, and individual metabolite-levels listed in separate rows.
(XLSX)

S4 Dataset Lipid data. Subject-index is listed in the first row, group in the second row, and individual lipid-levels listed in separate rows.
(XLSX)

S1 Fig Overview of MYBPC. Schematic drawing of MYBPC, indicating domains, myosin-binding region, titin-binding region, amino acid positions, and location of the *MYBPC3*-Q1061X mutation.
(TIF)

S1 Methods Lipidomic and metabolomic methods. (DOC)

S1 Table Metabolite-levels for significant molecular lipids and polar metabolites after adjusting for age and gender. (DOC)
