# Supplementary figures and images for: The Metabolome in Finnish Carriers of the MYBPC3-Q1061X Mutation for Hypertrophic Cardiomyopathy
Source: PLoS One. 2015 Aug 12;10(8):e0134184. doi: 10.1371/journal.pone.0134184 (PMC4534205; doi:10.1371/journal.pone.0134184)

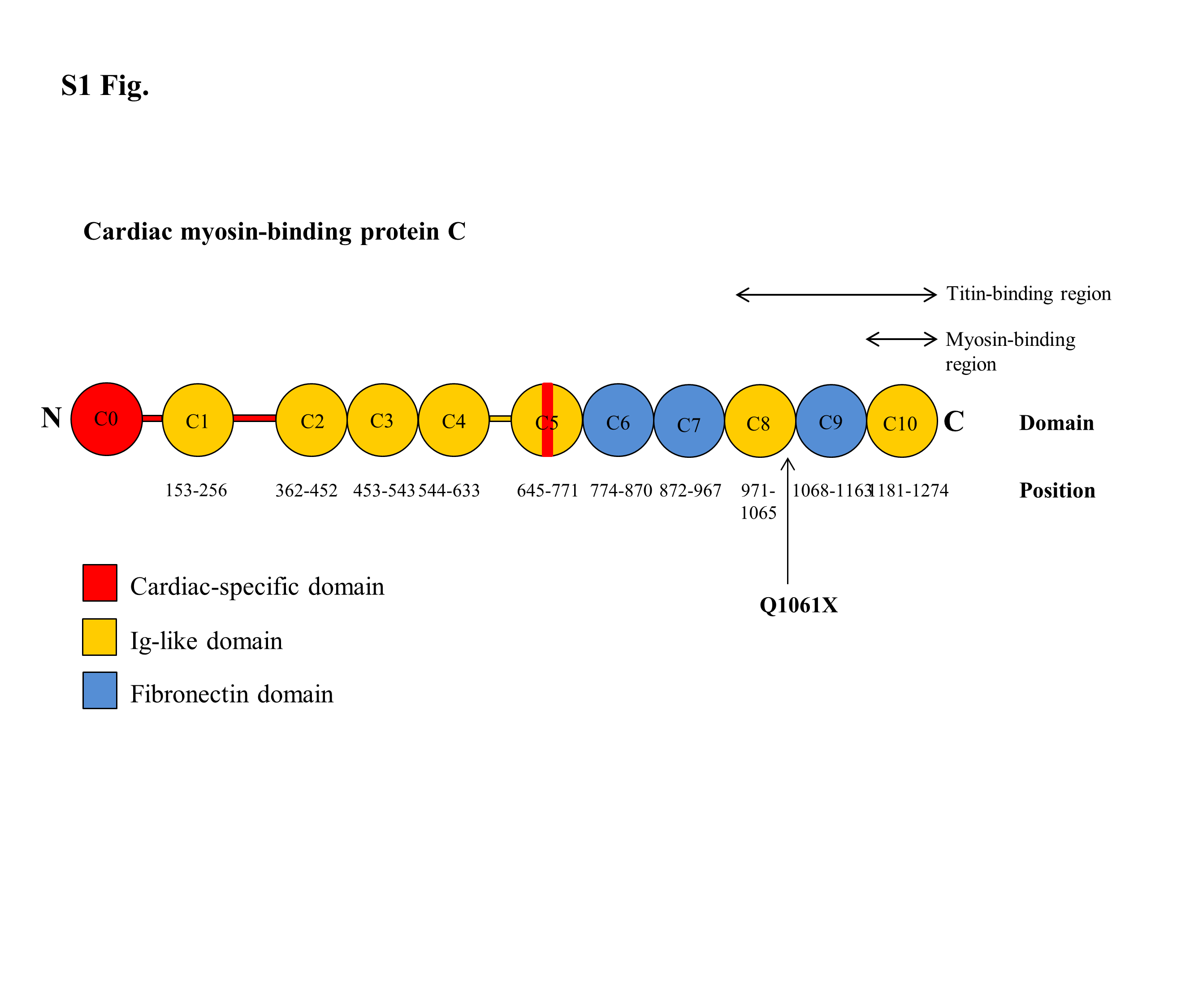

Supplement: S1 Fig — Schematic drawing of MYBPC, indicating domains, myosin-binding region, titin-binding region, amino acid positions, and location of the MYBPC3-Q1061X mutation. (TIF) [file pone.0134184.s005.tif]
