# Supplementary material for: The Metabolome in Finnish Carriers of the MYBPC3-Q1061X Mutation for Hypertrophic Cardiomyopathy
Source: PLoS One. 2015 Aug 12;10(8):e0134184. doi: 10.1371/journal.pone.0134184 (PMC4534205; doi:10.1371/journal.pone.0134184)
Supplement: S1 Methods — (DOC) [file pone.0134184.s006.doc]

## S1 Methods

**Lipidomics analysis.** For lipidomics analysis 10 µl of the plasma samples were mixed with 10 µl 0.9% (0.15 M) sodium chloride spiked with an internal standard mixture consisting of 10 lipids (0.2 µg/sample; lysoPC(17:0/0:0), PC(17:0/17:0), PE(17:0/17:0), PG(17:0/17:0), Cer(d18:1/17:0), PS(17:0/17:0), PA(17:0/17:0), MG/17:0/0:0/0:0)[rac], DG(17:0/17:0/0:0)[rac], TG(17:0/17:0/17:0, and CE(19:0)), where lysoPC is lysophosphatidylcholine, PC is phosphatidylcholine, PE is phosphatidylethanolamine, PG is phosphatidylglycerol, Cer is ceramide, PS is phosphatidylserine, PA is phosphatidic acid, MG is monoglyceride, DG is diglyceride, TG is triglyceride, and CE is cholesteryl ester). The samples were then extracted using 100 µl chloroform:methanol (2:1), vortexted for 2 min and left standing for 1 hour. After centrifugation at 10 000 revolutions per minute (rpm) (7826 G) for 3 min at room temperature, 60 µl of the lower organic phase was transferred to a new tube, and spiked with a standard mixture containing three labelled lipids (0.1 µg/sample; PC(16:0/0:0-D3), PC(16:0/16:0-D6) and TG(16:0/16:0/16:0-12C3)). In addition to the plasma samples standards, controls and blank samples were extracted.

The samples were run on an Acquity UltraPerformance LC™ system (UPLC) with an Acquity Sample Organizer, and analysed on a Waters QTOF Premiere mass spectrometer (Waters Corporation, Milford, MA, USA). The UPLC column was an Acquity UPLC™ BEH C18 1x50 mm with 1.7 µm particles, and had a temperature of 50°C. Buffer A consisted of 1% 1 M NH_4_Ac and 0.1% HCOOH in water, and buffer B consisted of acetonitrile/2-isopropanol (1:1, v/v), 1% 1 M NH_4_Ac and 0.1% HCOOH. The gradient started from 65% A-35% B, reached 100% B in 7 min, and stayed there for 7.5 min, and used 3.5 min for equilibration before next run. The flow rate was 0.400 ml/min, and 1 µl of each sample was injected. Reserpine was used as the lock spray reference compound. The profiling was carried out using ESI+ mode and the data were collected at a mass range of m/z 300 to 1200 (scan time 0.2 sec).

The analytical data were processed using the open-source software MZmine 2^1^, and subsequent identification and normalization was done using the open-source software Guineu^2^ with an internally acquired spectral library.

**Metabolomics analysis.** For analysis of small, polar metabolites, 30 µl of plasma from each of the subjects was spiked with 10 µl of internal standard (186.5 mg/l heptadecanoic acid, 37 mg/l Valine-d and 62.915 mg/l succinic acid-d4) and extracted with 400 µl of methanol. Labelled d-valine (37 mg/l) was added to the extracts as a derivatization standard. After centrifugation at 10 000 rpm for 5 min, the supernatant was evaporated to dryness, and the metabolites were derivatized in two separate steps. In the first step, 25 µl of methoxyamine hydrochloride (MOX) reagent was added to the supernatant, and incubated for 60 min at 45°C. During the second step 25 µl of N-methyl-N(trimethylsilyl)trifluoroacetatmide was added, and the mixture was incubated for 60 min at 45°C. The derivatized samples were then diluted 1:1 with hexane, and a retention index standard mixture (n-alkanes) and an injection standard (4,4’-dibromooctafluorobiphenyl), both in pyridine, were added to the mixture.

The samples were analysed on a Leco Pegasus 4D GCxGC-TofMS instrument (Leco Corp., St Joseph, MI, USA) equipped with a cryogenic modulator. The GC instrument used was an Agilent 6890N gas chromatograph (Agilent Technologies, Palo Alto, CA, USA), with a split/splitless injector. A pulsed splitless injection (0.5 µl) at 240°C was used, with pulse pressure of 55 psig for 1 min. The chromatographic column used in the first dimension was a 10-m RTX-5 capillary column with an internal diameter of 0.18 mm and a stationary-phase film thickness of 0.20 µm, while the second dimension was a 1.5-m BPX-50 capillary column with an internal diameter of 100 µm and a film thickness of 0.1 µm. A diphenyltetramethyldisilyl deactivated retention gap (2 m x 0.53 mm internal diameter) was used in front of the first column. High-purity helium was used as the carrier gas at a constant pressure mode (39.6 psig). A 5-sec separation time was used in the second dimension. The MS spectra were measured at 45 to 700 atomic mass unit with 100 spectra per second. Pulsed splitless injection 0.5 µl at 240°C was used. The temperature program was as follows: the first-dimension column oven ramp began at 40°C with a 2-min hold, after which the temperature was programmed to 295°C at a rate of 7°C/min and then held at this temperature for 3 min; the second-dimension column temperature was maintained 20°C higher than the corresponding first-dimension column. The programming rate and hold times were the same for both columns. The analytical data were processed using the Leco Chromatof and the open-source software Guineu^2^.

**Supporting References**

1. Pluskal T, Castillo S, Villar-Briones A, Orešič M. MZmine 2: Modular framework for processing, visualizing, and analyzing mass spectrometry-based molecular profile data. *BMC Bioinformatics*. 2010;11:395-2105-11-395. doi: 10.1186/1471-2105-11-395; 10.1186/1471-2105-11-395.

2. Castillo S, Mattila I, Miettinen J, Orešič M, Hyötyläinen T. Data analysis tool for comprehensive two-dimensional gas chromatography/time-of-flight mass spectrometry. *Anal Chem*. 2011;83(8):3058-3067. doi: 10.1021/ac103308x; 10.1021/ac103308x.
