# Supplementary material for: The Metabolome in Finnish Carriers of the MYBPC3-Q1061X Mutation for Hypertrophic Cardiomyopathy
Source: PLoS One. 2015 Aug 12;10(8):e0134184. doi: 10.1371/journal.pone.0134184 (PMC4534205; doi:10.1371/journal.pone.0134184)
Supplement: S1 Table — (DOC) [file pone.0134184.s007.doc]

**S1 Table: Metabolite-levels for significant molecular lipids and polar metabolites after adjusting for age and gender**

| Metabolite-name | G-/LVH- | G+/LVH- | G+/LVH+ | P-value |
| --- | --- | --- | --- | --- |
| PC(32:0) | -0.83 | 0.68 | 0.73^a,b^ | 0.006 |
| PC(32:2) | -0.47 | 0.05 | 0.41^a^ | 0.028 |
| PC(34:3) | -1.25 | 0.24 | 1.01^a^ | 0.037 |
| PC(36:4) | -12.04 | 14.23 | 11.49^a,b^ | 0.022 |
| PC(36:4e) | -0.08 | 0.05 | 0.07^a^ | 0.031 |
| PC(38:4) | -0.82 | 0.43 | 0.68^a^ | 0.049 |
| PC(40:4) | -0.03 | 0.03 | 0.03^a,b^ | 0.016 |
| PE(34:2) | -0.51 | 0.21 | 0.32^a^ | 0.018 |
| PE(36:4) | -0.38 | 0.33 | 0.18^b^ | 0.020 |
| PE(38:4) | -0.80 | 0.82 | 0.49^b^ | 0.026 |
| TG(49:2) | -0.03 | 0.03 | 0.03 | 0.039 |
| TG(58:5) | -0.01 | 0.01 | 0.01^a^ | 0.037 |
| TG(58:7) | -0.20 | 0.22 | 0.08^b^ | 0.020 |
| LysoPC(14:0) | -0.09 | 0.07 | 0.08^a^ | 0.043 |
| 4-Methyl-2-oxovaleric acid | -30.73 | 63.76 | 55.38 | 0.161 |
| Valine | -24.86 | 50.24 | 33.03 | 0.060 |
| Isoleucine | -10.18 | 29.11 | 10.28^b^ | 0.053 |
| Leucine | -24.42 | 56.54 | 28.72 | 0.059 |

The metabolite-levels are referred to as mean after adjusting for age and gender. Abbreviations: PE phosphatidylethanolamine, PC phosphatidylcholine, TG triglyceride, lysoPC lysophosphatidylcholine. P-values are given as one-way ANOVA calculated on the adjusted data. Pairwise comparisons with Tukey’s range test corrected p-values are labeled:

^a^P<0.05 05 between G+/LVH+ and G-/LVH-, given as Tukey’s range test.

^b^P<0.05 between G+/LVH- and G-/LVH-, given as Tukey’s range test.
